# Characterizing the Intra-Vineyard Variation of Soil Bacterial and Fungal Communities

**DOI:** 10.3389/fmicb.2019.01239

**Published:** 2019-05-31

**Authors:** Hebin Liang, Xiaowen Wang, Junwei Yan, Lixin Luo

**Affiliations:** ^1^School of Biology and Biological Engineering, South China University of Technology, Guangzhou, China; ^2^Food Testing Institute, Shenzhen Academy of Metrology and Quality Inspection, Shenzhen, China; ^3^National Nutrition Food Testing Center, Shenzhen, China

**Keywords:** vineyard soil, soil characteristics, intra-vineyard scale, bacterial community, fungal community

## Abstract

Vineyard soil microbial communities potentially mediate grapevine growth, grape production as well as wine *terroir*. Simultaneously assessing shifts of microbial community composition at the intra-vineyard scale allows us to decouple correlations among environmental variables, thus providing insights into vineyard management. Here we investigated bacterial and fungal community compositions and their relationships with edaphic properties in soils collected from a commercial vineyard at four different soil depths (0–5, 5–10, 10–20, and 20–40 cm). Soil organic carbon (SOC) content, invertase activity and phosphatase activity decreased along depth gradient in the 0–20 cm soil fraction (*P* < 0.001). The soil bacterial biomass and α-diversity were significantly higher than those of fungi (*P* ≤ 0.001). Statistical analyses revealed that SOC content, pH, C/N ratio and total phosphorus (TP) were significant determinants of soil bacterial (*R* = 0.494, *P* = 0.001) and fungal (*R* = 0.443, *P* = 0.001) community structure. The abundance of dominated bacterial phyla (*Proteobacteria*, *Acidobacteria* and *Actinobacteria*) and fungal phyla (*Ascomycota*, *Zygomycota* and *Basidiomycota*) slightly varied among all soil samples. Genus *Lactococcus*, which comprised 2.72% of the soil bacterial community, showed increasing pattern with depth. Importantly, *Candidatus Nitrososphaera*, *Monographella* and *Fusarium* were also detected with high abundances in soil samples, indicating their ecological function in soil nitrogen cycle and the potential risk in grapevine disease. Overall, this work detected the intra-vineyard variation of bacterial and fungal communities and their relationships with soil characteristics, which was beneficial to vineyard soil management and grapevine disease prevention.

## Introduction

Soil is a pivotal component of the ecosystem and generally acts as a microbial reservoir for plants, especially concerning underground plant microbe (Corneo et al., 2013; Mezzasalma et al., 2018). Complex physiochemical and biochemical interactions are crucial to soil quality, where biochemical process is a vital process in maintaining ecosystem equilibrium and affects the links between plants and macroscopic environmental conditions (Corneo et al., 2013; Canfora et al., 2018; Mezzasalma et al., 2018). There is a complex web of microorganisms in soil and their diversity and composition vary in space and time (Canfora et al., 2018). Vineyard soil microorganisms play critical roles in grapevine growth, productive capacity and wine *terroir* formation (Bokulich et al., 2014). Hence, gaining a thorough understanding of the microbial distribution in vineyard soil and complex linkages among microbial consortia, soil characteristics and environmental conditions may provide new insights in improvement of grape production and vineyard management practices.

Soil microbial consortia can affect plant growth through direct and indirect mechanisms (Mendes et al., 2013; Plaza et al., 2013; Zarraonaindia et al., 2015; Burns et al., 2016). For instance, organo-mineral complexes formed by soil microorganisms mainly contribute to the long-term organic matter stabilization and carbon sequestration (Plaza et al., 2013). In addition, some growth-promoting bacteria, such as *Pseudomonas*, *Bacillus*, *Streptomyces* and *Micromonospora*, can colonize root as rhizobacteria to confer pathogen resistance or improve productivity (Loqman et al., 2009; Martins et al., 2013; Mendes et al., 2013). Moreover, the members of phylum *Proteobacteria*, such as *Steroidobacter*, *Bradyrhizobium* and *Rhizobium*, are beneficial to plant development and physiology due to the excretive brassinosteroids and nitrogen fixation (Yin-Ru Chiang, 2011; Mendes et al., 2013). Recent studies suggested that the organ-associated phylotypes mostly derive from soil and migration of microorganisms from soil can potentially affect the regional patterns in wine chemosensory properties (Bokulich et al., 2014; Zarraonaindia et al., 2015; Mezzasalma et al., 2018). Thus, investigation of soil-borne microbial community distributions is an important aspect of soil quality evaluation and sustainable vineyard management (Mendes et al., 2013; Burns et al., 2016). In the past decades, the bacterial communities associated with vineyard soil and grapevine organs have been well documented for their potential biotechnological applications (Fernández-Calviño et al., 2010; Martins et al., 2013; Zarraonaindia et al., 2015). The bacterial community composition of the vineyard soil ecosystem is generally predominated by phyla *Proteobacteria*, followed by *Actinobacteria*, *Bacteroidetes*, *Verrucomicrobia* and *Firmicutes* (Eilers et al., 2012; Zarraonaindia et al., 2015; Mezzasalma et al., 2018). Fungi were generally considered as plant pathogens for causing substantial economic damage in grape production (Mendes et al., 2013), while they could also act as mycorrhizal mutualists and decomposers of several recalcitrant compounds in soil, such as cellulose, hemicellulose and lignin (Orgiazzi et al., 2012). Likewise, oenologically important yeast, like *Saccharomyces cerevisiae* which is essential for alcoholic fermentation, has been isolated from vineyard soil (Martins et al., 2013). However, previous studies mostly focused on the bacterial community in vineyard ecosystem, while there is dearth of information on fungal diversity in vineyard soil as well as their complex interactions with bacteria and plants (Orgiazzi et al., 2012; Holland et al., 2016).

Nevertheless, soil-borne microbial community composition is generally influenced by myriad factors, such as soil properties (Allison et al., 2007; Steenwerth et al., 2008; Liu et al., 2014), depth (Steenwerth et al., 2008; Eilers et al., 2012), agronomic practices (Burns et al., 2016; Canfora et al., 2018), altitudinal gradients (Corneo et al., 2013; Chang et al., 2016) and climates (Burns et al., 2015). Previous literatures have demonstrated that variations in microbial community structures are associated with the altitude, which is considered as a complex physicochemical gradient (Corneo et al., 2013; Chang et al., 2016). However, local environmental heterogeneity can also drive soil bacterial biogeographical patterns or even outweigh biogeographic trends (Zarraonaindia et al., 2015). Additionally, the work by Burns et al. (2015) also demonstrate that the soil bacterial diversities within the same vineyard still have great phylogenetic distances. Therefore, we investigated physicochemical characteristics, enzyme activities and microbial community composition in vineyard soils, which were collected from four sites and depths in a grapevine growing region. The present study aimed to: (1) simultaneously characterize the bacterial and fungal communities in vineyard soils, and (2) investigate the relationships between environmental factors and microbial community. Altogether, this work might provide new insights into vineyard soil management and grapevine disease prevention.

## Materials and Methods

### Site Description and Soil Sampling

Soil samples were taken from a commercial vineyard, which cultivated *Vitis davidii* in the north of Tongren city, west of Guizhou province, southwest China (109°35′E, 27°49′N). Vine plantation was organized with 2.5 m × 0.9 m distance between rows and plants. The site is characterized by a subtropical wet monsoon climate, with a mean annual temperature of 15.8–17.3°C and an average annual precipitation of approximately 1156.4–1432.9 mm.

In this vineyard, 4 soil plots each separated from the other by 20 m were randomly selected at approximately 25 cm away from the vine rows. After removing the residues on the surface, a total of 16 soil samples were collected from a depth of 0–5, 5–10, 10–20, and 20–40 cm at each plot with a handle steel soil sampler in this grape vineyard to ensure the similar climatic condition and viticulture management. At each sampling site, three soil samples were collected and mixed into a composite sample. Homogenized soil was immediately placed in sterile bags and transported to the laboratory on ice for laboratory analyses. After removal of plant residues and shoots, all the samples were divided into two subsamples, one each for soil chemistry and microbial community analysis. The subsample was air dried in the laboratory for 2 days and sieved through 2.0 mm mesh before soil chemistry analysis.

### Soil Chemical and Biochemical Analyses

Soil physicochemical characteristics including pH, moisture, soil organic carbon (SOC), total nitrogen (TN), total phosphorus (TP) and available phosphorus (AP), were determined in each sample. Soil pH was measured in a soil and distilled water suspension (1:5 w/v) using a PB-10 pH meter (Sartorius, Germany) after 30 min shaking. Soil moisture content was determined after oven-drying samples for 24 h at 105°C. SOC, TN, TP, and AP content were correspondingly determined by potassium dichromate volumetric method, Kjeldahl method, HClO_4_–H_2_SO_4_ fusion method and NaHCO_3_ method (Gasparatos and Haidouti, 2001; Qi et al., 2016).

The soil amylase and invertase (EC 3.2.1.26) activities were measured by 3,5-dinitrosalicylic acid method as described by Deng and Tabatabai (1994). Catalase activity (EC 1.11.1.6) was determined as described by Cohen et al. (1970). Urease activity (EC 3.5.1.5) was estimated determined as described by Kandeler and Gerber (1988). Phosphatase activity (EC 3.1.3.2) was measured as described by Tabatabai and Bremner (1969).

### DNA Extraction and Sequencing

Total genomic DNA was extracted using 0.4 g air-dried soil and the Soil DNA Kit (Omega Bio-Tek, Norcross, GA, United States) according to the manufacturer’s instructions. Quality of the extracts was assessed using gel electrophoresis and a NanoDrop spectrophotometer (Thermo Fisher Scientific, DE, United States) and then stored at -80°C for further analysis.

Amplification and sequencing were performed as described previously for analysis of bacterial (Caporaso et al., 2011) and fungal communities (Huang et al., 2016). Briefly, the V4 region of bacterial 16S rRNA genes was amplified in triplicate for each sample using primers 515F/806R with 6-nt barcode as described by Caporaso et al. (2011). The polymerase chain reaction (PCR) was performed under the following conditions: initial denaturation at 95°C for 2 min; 30 cycles at 95°C for 15 s, 55°C for 15 s, and 72°C for 30 s; and a final extension at 72°C for 10 min. Fungal internal transcribed spacer (ITS) 1 region was amplified with primers ITS5-1737F/ITS2-2043R (ITS5-1737F: GGAAGTAAAAGTCGTAACAAGG; ITS2-2043R: GCTGCGTTCTTCATCGATGC) with 6-nt barcode as described by Huang et al. (2016). PCR conditions consisted of initial 98°C for 1 min, followed by 30 cycles of 98°C for 10 s, 50°C for 30 s, and 72°C for 60 s as well as a final extension of 72°C for 5 min. Amplicons for each sample were purified with GeneJET Gel Extraction Kit (Thermo Fisher Scientific) and then pooled in equal molar quantities for library construction. Libraries were prepared using TruSeq^®^DNA PCR-Free Sample Preparation Kit (Illumina, United States). After assessment of the libraries by Qubit@ 2.0 Fluorometer (Thermo Fisher Scientific, MA, United States) and Agilent Bioanalyzer system, the libraries were sequenced on an Illumina HiSeq 2500 platform (Beijing Novogene Bioinformatics Technology Co., Ltd., Beijing, China).

### Quantitative PCR

Quantification of 16S rRNA genes and fungal ITS fragment was carried out with an ABI 7500 Real Time PCR System (Applied Biosystems, Germany), and mixture commercial kit (SYBR Premix Ex Taq, Takara, Japan). Standard curves were generated using tenfold dilutions of plasmid qRT-11 (KX350063) containing the target region of 16S rRNA gene and plasmid Y-qRT-1 (KM492830) containing the partial 18S rRNA gene fragments. The amplification reactions were conducted with primers P1/P2 for bacteria and Y1/Y2 for fungi (Muyzer et al., 1993; Xu et al., 2011). PCR reactions and conditions were performed following our previous study (Li et al., 2017). All the samples and the no-template control were analyzed in triplicate. PCR efficiency values for the abundance of 16S rRNA and 18S rRNA were 99.50% (*R*^2^ = 0.997) and 99.09% (*R*^2^ = 0.993), respectively.

### Data Analyses

The sequence data were extracted based on the unique sample barcodes, trimmed for sequence quality, and then denoised using QIIME v1.7.0 (Caporaso et al., 2010). Operational taxonomic units (OTUs) were assigned with a threshold of 97% pairwise identity and classified as described by Bokulich et al. (2014). α-diversity estimates were calculated by analyzing the observed species, Shannon index, Simpson index and Chao1 richness. Weighted UniFrac-based principal coordinates analysis (PCoA) plots and cluster analysis were used to examine the between-sample community dissimilarity. Permutational multivariate analysis of variance (PERMANOVA) with 999 permutations were used to test significant differences between different sampling depths as well as sites. One-way analysis of variance (ANOVA) was performed with Duncan’s test to evaluate significant difference (*P* < 0.05) in soil physicochemical properties (moisture, pH, SOC, TN, TP, AP) and enzyme activities among different soil depths in each site (Qi et al., 2016). Mantel test was used to determine the associations among soil edaphic factors and microbial community structures. Then, the soil properties affecting the microbial consortia were further explore by the Bray–Curtis distance-based redundancy analysis (db-RDA) using Canoco 5.0 software. Subsequently, a best variables rank correlation test (BEST) was used to rank the importance of edaphic properties in influencing β-diversity community comparisons and identify the ones capturing the greatest variance in the community. Spearman correlation coefficient (SPCC) was calculated to estimate the significant correlations of physicochemical properties, enzymatic activities and soil microbial community composition and abundance. The strong and significant correlations (SPCC ≥ 0.75 or ≤ –0.75; corrected *P*-value ≤ 0.05) were visualized in heatmap.

### Accession Numbers

Raw data in this study have been deposited in the NCBI Short Read Archive database under accession numbers SRP2085086 (16S) and SRP2085088 (ITS).

## Results

### Soil Physicochemical Properties and Enzyme Activities

Soil moisture varied from 23.32 to 13.32% and gradually decreased to a minimum near 20 cm apart from soils in site C where moisture was higher in 5–10 cm (Table 1). However, there was no significant differences among the sampling sites (*P* > 0.84). Vineyard soils in this work were all alkalescent and no significant differences in pH were measured for either different depths or sampling sites (Table 1, *P* > 0.05). The SOC, TN, and TP slightly decreased with depth in the top 20 cm and then increased in the 20–40 cm soil profiles (Table 1). Notably, SOC contents showed a significant downtrend in the top 20 cm soil fraction (*P* < 0.001), meanwhile, SOC contents in site C were significantly higher than other sites (Table 1, *P* < 0.001). In addition, AP contents statistically decreased with increasing soil depth (*P* < 0.01) but no detectable differences across four sites (*P* > 0.1).

**Table 1:** The chemical and physical properties of different soil samples.

Sample	Site	Depth (cm)	Moisture (%)	pH	SOC (g/kg)	TN (mg/kg)	C/N	TP (g/kg)	AP (mg/kg)
A1.5	A	0–5	20.29	8.14	27.71 ± 0.47	1.78 ± 0.12	15.60	0.98 ± 0.13	28.42 ± 0.44
A1.10		5–10	17.97	8.13	22.22 ± 0.67	1.50 ± 0.11	14.86	0.81 ± 0.03	18.93 ± 0.57
A1.20		10–20	15.15	8.05	15.24 ± 0.26	1.26 ± 0.53	12.06	0.65 ± 0.05	8.07 ± 1.99
A1.40		20–40	17.82	8.19	17.08 ± 1.19	1.67 ± 0.24	10.21	0.75 ± 0.02	4.36 ± 0.59
B2.5	B	0–5	23.32	8.05	25.15 ± 0.15	1.9 ± 0.28	13.26	1.06 ± 0.05	19.74 ± 2.06
B2.10		5–10	17.50	8.10	19.99 ± 0.83	1.36 ± 0.19	14.66	0.94 ± 0.03	11.78 ± 0.68
B2.20		10–20	17.22	8.07	18.38 ± 0.36	1.14 ± 0.14	16.16	0.8 ± 0.07	10.90 ± 0.57
B2.40		20–40	17.47	8.02	19.13 ± 0.81	1.46 ± 0.17	13.11	0.9 ± 0.02	8.93 ± 0.48
C3.5	C	0–5	18.01	7.92	41.78 ± 0.11	2.22 ± 0.57	18.82	0.91 ± 0.01	24.99 ± 0.61
C3.10		5–10	18.87	7.88	33.79 ± 2.27	2.10 ± 0.05	16.09	0.82 ± 0.04	16.15 ± 3.54
C3.20		10–20	17.56	7.95	28.34 ± 0.48	1.89 ± 0.18	14.96	0.58 ± 0.11	9.79 ± 1.25
C3.40		20–40	19.91	7.90	30.56 ± 0.04	2.03 ± 0.10	15.08	0.70 ± 0.02	9.47 ± 1.00
D4.5	D	0–5	21.80	7.88	33.87 ± 0.06	2.10 ± 0.26	16.14	0.76 ± 0.07	39.73 ± 0.81
D4.10		5–10	18.74	7.86	27.95 ± 0.50	1.82 ± 0.19	15.35	0.60 ± 0.02	21.39 ± 1.65
D4.20		10–20	13.32	7.95	16.16 ± 0.24	1.01 ± 0.05	16.07	0.58 ± 0.01	8.09 ± 0.62
D4.40		20–40	15.61	7.93	15.31 ± 0.17	1.08 ± 0.15	14.11	0.58 ± 0.01	5.48 ± 0.65


Soil amylase activity ranged from 1.89 to 5.62 mg maltose g^-1^ 24 h^-1^ and reached a maximum activity at 5–10 cm depth in all sites (Supplementary Table S1), whereas there was no difference in the amylase activity across the four sites (*P* > 0.16). Soil invertase activity and phosphatase activity varied from 0.45 to 7.18 mg glucose g^-1^ 24 h^-1^ and from 1.82 to 29.81 mg phenol 100 g^-1^ 24 h^-1^, respectively. Meanwhile, both invertase activity and phosphatase activity decreased with increasing depth. The invertase activity in sites B and C soils was statistically greater than that in sites A and D soils in the 10–40 cm soil fraction (Supplementary Table S1, *P* < 0.01). Urease activity stabilized in the range of 0.12–0.17 mg NH_4_^+^–N g^-1^ 24 h^-1^ across all samples (Supplementary Table S1, *P* > 0.05). Similarly, apart from the 10–20 cm soil profile of site D (4.34 ± 0.74 0.02 M KMnO_4_ ml g^-1^), there was no significant change in catalase activity in subsoils for any of the depths and sites (Supplementary Table S1, *P* > 0.05).

### Microbial Abundance, Diversity, and Composition in Vineyard Soils

#### Abundances of Bacteria and Fungi in Vineyard Soils

In this study, the abundances of total bacterial 16S rRNA and fungal 18S rRNA genes in the 0–40 cm soil profiles were analyzed by quantitative PCR. The bacterial and fungal biomass varied from 9.57 to 10.03 log 10 copies/g and from 5.97 to 6.91 log 10 copies/g in the 0–40 cm soil profiles, respectively (Supplementary Figure S1). Bacterial biomass slightly declined with the depth in all sites and were about 1000-fold higher than fungi (Supplementary Figure S1). Moreover, fungal biomass slightly decreased in the 0–20 cm soil profiles. Nevertheless, both bacterial and fungal biomass among four sites exhibited no marked differences (Supplementary Figure S1, *P* > 0.05).

#### Microbial Diversity Patterns in Vineyard Soils

**FIGURE 1:**
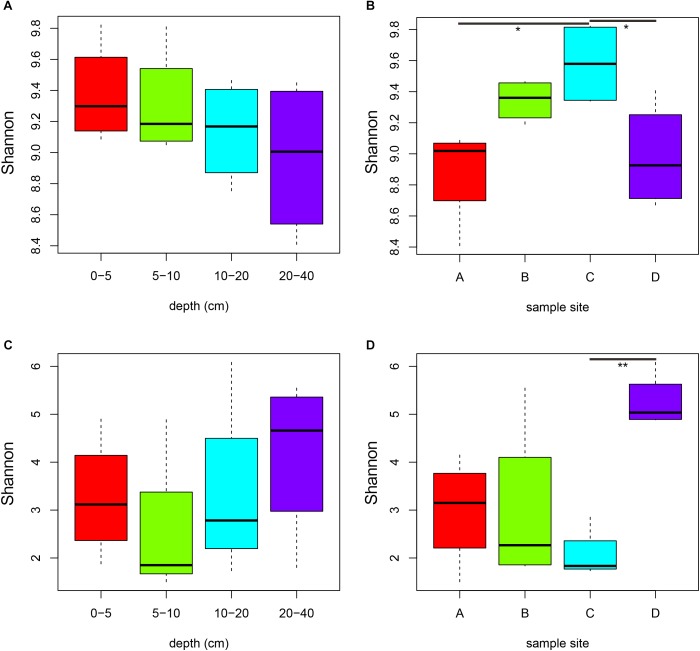
Comparisons of bacterial **(A,B)** and fungal **(C,D)** Shannon diversity indexes among soil depths **(A,C)** and among sites **(B,D)**. Significance: ^∗^*P* < 0.05, ^∗∗^*P* < 0.01.

In profiling the sequencing data, a total of 843,149 bacterial 16S rRNA gene tags and 939,525 fungal ITS1 sequence tags were generated after quality control (Supplementary Table S2). At 97% sequence identification, a total of 41,882 and 6,567 OTUs were detected for bacteria and fungi, respectively (Supplementary Table S2). The rarefaction curves tended to approach the plateau phase for all samples, suggesting that the sequencing depth was sufficient to capture the majority of the microbial diversity (Supplementary Figure S2). In all samples, higher number of bacterial OTUs, Chao1 estimator and Shannon index were observed than those of fungi (Figure 1, Supplementary Figure S3 and Supplementary Table S2, *P* < 0.001). Both bacterial and fungal diversity showed remarkably distinctions among the four sites (Figure 1B,D, *P* < 0.05). For the bacterial community, the Shannon diversity slightly decreased with increasing depth (Figure 1A). Bacterial Shannon indices of samples in site C were significantly higher than samples from sites B and D (*P* < 0.05), whereas fungal diversity of soils from site D was obviously higher than samples from site C (Figure 1D, *P* = 0.007).

#### Microbial Community Structures in Vineyard Soils

**FIGURE 2:**
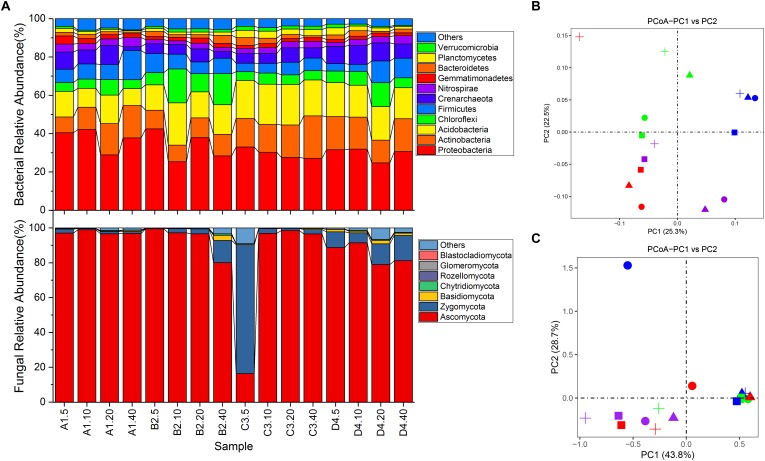
Differences in microbial community structures and compositions. **(A)** relative abundance of microbial community composition characterized to the phylum level (top 10 taxa). **(B)** principle coordination analysis (PCoA) of bacterial community among all soil samples. **(C)** PCoA of fungal community among all soil samples. Shapes in circle, triangle, square and cross represent samples from site A, B, C and D, respectively. Shapes in color red, green, blue and purple represent samples from 0 to 5 cm, 5 to 10 cm, 10 to 20 cm and 20 to 40 cm, respectively.

An overview of bacterial succession is reported in Figure 2A. *Proteobacteria* dominated in all soils (24.72–42.47%, averaging at 32.51%), followed by *Acidobacteria* (8.81–22.11%, averaging at 16.26%), *Actinobacteria* (8.21–22.16%, averaging at 14.02%), *Chloroflexi* (4.56–17.64%, averaging at 7.69%) and *Firmicutes* (3.74–15.29%, averaging at 7.63%) (Figure 2A). Strikingly, two archaeal phyla, *Crenarchaeota* (2.01–9.96%, averaging at 6.71%) and *Euryarchaeota* (0–0.4%, averaging at 0.1%) were also detected in each soil sample. PCoA showed that the distribution of bacterial communities was not clearly separated by either depth or sites (Figure 2B). Cluster analysis revealed that the 16 samples were clustered into 4 groups (Supplementary Figure S4A). It was shown that samples from sites A, B, and D were complicatedly related, while 4 samples from site C were closely related (Supplementary Figure S4A). Nevertheless, results of PERMANOVA analysis showed no statistically significant differences in soil bacterial community composition for any of the depths (*P* > 0.51) and sites (*P* > 0.12). However, the relative abundance of individual bacterial taxa varied in different depths and sample sites. For instance, taxa *Acidobacteria-6* tended to be more abundant in the 0–20 cm soil profiles, whereas *Bacilli* and *Alphaproteobacteria* were more common in 10–40 cm and 0–10 cm soil fractions, respectively (Supplementary Figure S5A). Meanwhile, the relative abundance of *Betaproteobacteria* in site A was much higher than other samples (Supplementary Figure S5A). In addition, soils from site A and B contained more *Lactococcus* than *Arthrobacter*, while the soils from C and D exhibited the converse tendency (Supplementary Figure S5B). Similarly, genus *Pseudomonas* was detected with low abundance in soils from site C than soils from other sites (Supplementary Figure S5B, *P* < 0.13). Genus *Lactococcus* was proportionally abundant in soils at 20–40 cm except sample from site C (Supplementary Figure S5B).

As for fungal communities, members of the phylum *Ascomycota* dominated the eukaryotic community of all vineyard soils except sample C3.5 where *Zygomycota* was the most abundant taxa (74.03% of total effective sequences) (Figure 2A). Phylum *Basidiomycota* was only the major taxa (relative abundance > 1%) in samples B2.40, D4.5, D4.20 and D4.40. However, no marked differences were detected in the relative abundances of these phyla across all samples at different depths and sites (Figure 2A, *P* > 0.15). Furthermore, 59.86% of the effective tags were classified to 293 genera. Most of the abundant fungal genera including *Tetracladium*, *Monographella*, *Rhizomucor*, *Peziza*, *Fusarium*, *Chaetomium*, *Mortierella* and *Aspergillus*, showed a shift of relative abundance in different soils (Supplementary Figure S5C). Among them, members of *Tetracladium* (on average 28.18%) were more abundant in soils from sites B and C than the other two sites. Nevertheless, the structure of fungal community, similarly to what observed for bacterial community, showed no significant differences for any of the depths and sites (Figure 2C, *P* > 0.192). Cluster analysis further illustrated the complicated relationships among all the samples (Supplementary Figure S4B). In addition, only four genera including *Candida*, *Meyerozyma*, *Cyberlindnera* and *Wickerhamomyces* belonging to class *Saccharomycetes* were detected with low relative abundances in some samples (data not shown).

### Linkages Among Microbial Community, Enzyme Activities, and Soil Properties

**FIGURE 3:**
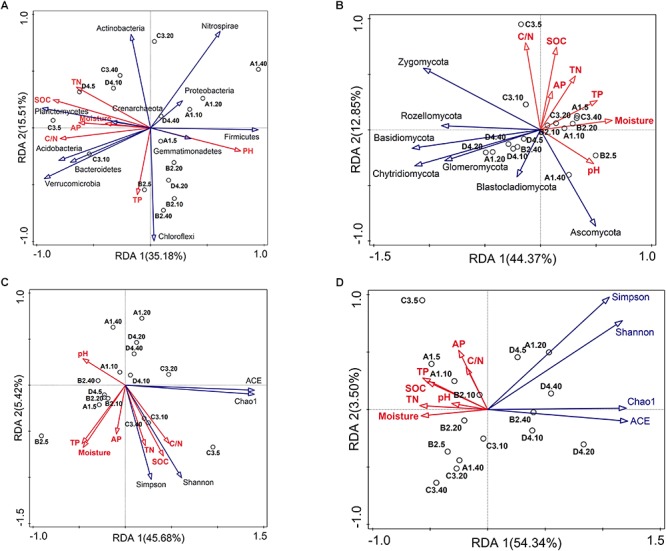
Bray–Curtis distance-based redundancy analysis (db-RDA) of the soil bacterial **(A,C)** and fungal **(B,D)** community and diversity constrained by soil characteristics.

Soil properties played pivotal roles in shaping bacterial and fungal communities [Mantel *R* = 0.321 (*P* = 0.007) and *R* = 0.339 (*P* = 0.006), respectively]. Results from BEST identified pH, SOC content, C/N ratio and TP as prevailing factors for explaining the soil bacterial community composition (*R* = 0.494, *P* = 0.001), whereas only SOC content explained the greatest variation of fungal community (*R* = 0.443, *P* = 0.001). Bray–Curtis distance-based redundancy analysis (db-RDA) between soil microbiota and physicochemical characteristics depicted the interdependence of microbial community composition and soil chemistry (Figure 3A,B). The large predominance of *Proteobacteria* was positively correlated with pH (Figure 3A, *P* < 0.05), while soil pH and TP were negatively related to the relative abundance of *Actinobacteria* (Figure 3A, *P* < 0.05). Soil SOC contents, TN, C/N ratio and AP possessed strong and remarkably positive correlations (SPCC ≥ 0.75, *P* < 0.05) with the relative abundances of *Verrucomicrobia*, *Planctomycetes* and *Bacteroidetes*, while negatively correlated with the relative abundance of *Firmicutes* (Figure 3A). Regarding fungal phyla, the most abundant phyla *Ascomycota* and *Zygomycota* showed the contrary correlations with soil texture, while phyla *Basidiomycota*, *Chytridiomycota*, *Rozellomycota*, *Glomeromycota*, *Chlorophyta* were negatively associated with soil characteristics (Figure 3B). Moreover, bacterial diversity was closely associated with soil SOC, TN and C/N (Figure 3C and Supplementary Table S3). Thereinto, bacterial Shannon index was positively related with the C/N (SPCC = 0.61, *P* < 0.05). In contrast to bacterial diversity, fungal diversity mostly kept negative relationships with soil properties, especially Chao1 estimator which showed significant negative correlations with the moisture, SOC, TN, TP and AP contents (Figure 3D and Supplementary Table S4, *P* < 0.05).

**FIGURE 4:**
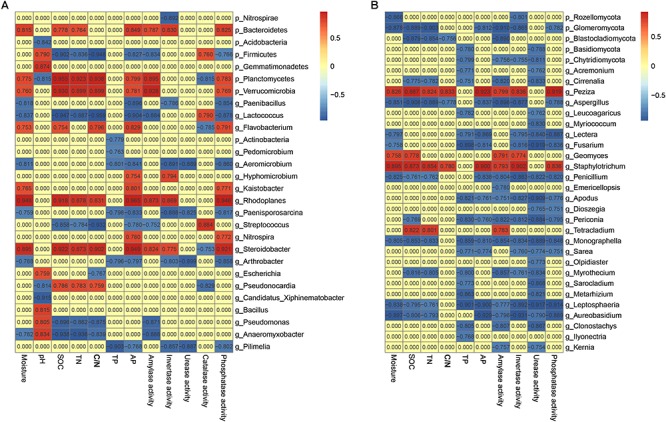
Heatmaps showing bacterial **(A)** and fungal **(B)** co-occurrence in soils. Top 10 bacterial phyla, top 7 fungal phyla as well as top 35 genera were displayed. A connection stands for SRCC with a magnitude >0.75 or < –0.75 and statistically significant (*P* < 0.05).

Keystone species are commonly defined as species with large ecological functions that were disproportionately relative to their abundance (Power et al., 1996). Correlation analysis was conducted to explore the co-occurrence patterns among soil chemistry, enzyme activities and keystone species in soils (Figure 4). Meanwhile, a total of 47 genera (abundance ≥ 0.01%) including 20 bacterial genera and 27 fungal genera was identified with significant associations with soil edaphic factors and enzyme activities (Figure 4). The correlation analysis identified bacterial genera *Lactococcus*, *Arthrobacter*, *Pseudomonas*, *Rhodoplanes*, *Bacillus* and *Nitrospira* and fungal genera *Tetracladium*, *Monographella*, *Fusarium*, *Peziza*, *Aspergillus* and *Kernia* as the keystone genera in these soil profiles. Of these, genus *Lactococcus* was negatively related with SOC contents (SPCC = –0.947, *P* < 0.001) and TN (SPCC = –0.887, *P* < 0.001). Additionally, *Pseudomonas* possessed strong and significantly negative correlations with SOC contents (SPCC = –0.896, *P* < 0.001) and TN (SPCC = –0.862, *P* < 0.001). Overall, most of bacterial keystone genera belonged to *Proteobacteria* (4.14% of total effective sequences) and *Firmicutes* (4.08%), followed by *Actinobacteria* (2.96%), *Bacteroidetes* (0.18%) and *Verrucomicrobia* (0.11%), while the fungal keystone genera affiliated to phyla *Ascomycota* (50.71%), *Basidiomycota* (0.29%) and *Chytridiomycota* (0.08%).

## Discussion

### Heterogeneity in Soil Chemistry and Enzyme Activity at Intra-Vineyard Scale

The formation of environmental heterogeneity is mostly caused by different geographic features (Liu et al., 2014; Burns et al., 2015), depth (Griffiths et al., 2003; Eilers et al., 2012), climate (Bokulich et al., 2014) and management (Burns et al., 2016; Canfora et al., 2018). In this work, the spatial variation of SOC contents was in the range of observed fir plantation soil (Chen et al., 2015) and vineyard soils (Probst et al., 2008; Burns et al., 2015). The significant decline of SOC contents at 0–20 cm depth was likely derived from the reduced microbial transformation of soil organic matter caused by somewhat lower water contents (Probst et al., 2008). Indeed, soil moisture decreased with depth in the 0–20 cm soil profiles, thereby agreeing with the results obtained in previous studies (Griffiths et al., 2003; Eilers et al., 2012; Chen et al., 2015). Moreover, the significant higher SOC contents in site C indicated environmental heterogeneity even appeared across small distance (Steenwerth et al., 2008; Zarraonaindia et al., 2015). The significant decrease in AP with depth was also observed in sandy and slit loam soils (Blume et al., 2002). The soil depth significantly associated with AP decrease was likely caused by the assimilation of grapevine root which generally distributed at deep layers (20–40 cm).

Enzyme activities can be potentially useful for evaluating soil quality, since they can participate in nutrient cycling and play significant psychological functions in maintaining soil structure, degradation pollutants, and producing essential compounds for microorganisms and plants. In the present study, both amylase and invertase activities were much higher in the upper 10 cm soils (Supplementary Table S1), which was consistent with previous studies in Brazilian Typic Haplorthox soil (de Melo et al., 2007), wetland system (Baddam et al., 2016) and vineyard soils (Miguéns et al., 2007). Nevertheless, the phosphatase activity obviously decreased with depth, which was in agreement with the significant downtrend of AP (Table 1) and the observation in wet land systems (Baddam et al., 2016).

### Changes in Microbial Biomass, Diversity and Dynamics

The slight decrease in bacterial biomass with depth has been manifested in previous studies in vineyard soil (Steenwerth et al., 2008), sandy and slit loams (Blume et al., 2002). Chen et al. (2015) identified that soil microbial biomass carbon and microbial biomass nitrogen were significantly higher in the surface soil (0–10 cm soil depth) than in subsurface soil (10–20 cm soil depth). Apparently, the bacterial biomass in this work did slightly decline with depth, consistent with previous work (Zhang et al., 2017). Moreover, both bacterial and fungal biomass were slightly higher in site C than other sites, which could be explained by the higher SOC contents in site C (Blume et al., 2002) (Table 1 and Supplementary Figure S1). In this regards, the results showed that soil depth and carbon heterogeneity could be regarded as ecological filters for shaping different intra- and inter-vineyard microbial biomass.

Overall, we found a general decrease in bacterial diversity (Shannon index) with increasing depth, which was in agreement with previous work by Zhang et al. (2017). Moreover, higher bacterial diversity (Figure 2A,B) and richness (Supplementary Figures S3A,B) in soils from site C was likely caused by the significantly higher SOC contents in site C (Table 1). It is known that bacteria are more sensitive than fungi to alteration of nutrient availability, as they proliferate faster than fungi and react faster to changes in soil nutrients (Li et al., 2019). However, bacterial communities in those soil samples inconspicuously varied with depth gradient (Figure 2B). Eilers et al. (2012) found that the soil microbial communities from the deeper soil horizons were most similar to the surface soils collected from a variety of temperate and tropical forest sites, which suggested that the magnitude of bacterial community changes with depth was equivalent to the magnitude of changes observed across surface samples collected from a wide range of geographic distances. Furthermore, fungal community similarity and cluster analysis showed no obvious differences among four depths and sites (Figure 2 and Supplementary Figures S3, S4B), which was probably interpreted by that the differences in soil characteristics among samples were not large enough to affect fungal community (Holland et al., 2016). Therefore, bacterial community was more complex and discrete than fungal community (Figure 2B,C)

Vineyard soil was considered as a complex ecosystem consisting of an intricate web of microorganisms which likely played pivotal roles in grapevine growth, grape production as well as following wine fermentation (Bokulich et al., 2014; Burns et al., 2015; Mezzasalma et al., 2018). The bacterial community in all soil samples were dominated by phyla *Proteobacteria*, *Acidobacteria* and *Actinobacteria* (Burns et al., 2015) determined that the dominant bacterial groups across vineyard soils in Napa Valley AVA were *Proteobacteria*, *Actinobacteria*, *Acidobacteria* and *Bacteroidetes*, which resembled with the present work. Moreover, bacterial affiliations in agricultural landscape soils were generally dominated by *Proteobacteria*, *Actinobacteria*, *Bacteroidetes*, *Acidobacteria* and *Firmicutes* (Constancias et al., 2015). Likewise, bacterial communities were mostly composed of *Proteobacteria*, *Actinobacteria* and *Acidobacteria* in Italy and Spain vineyard soils (Mezzasalma et al., 2018), suggesting that bacteria exhibited ecological coherence in vineyard soils even at coarse taxonomic levels. *Proteobacteria* are widespread in soil environments and are of great importance to carbon, nitrogen, and sulfur cycling (Spain et al., 2009). Previous work reported that *Burkholderiales* and *Rhodobacterales* within *Proteobacteria* involved in denitrification in paddy soil (Chen et al., 2010). Bacteria within *Acidobacteria* are ubiquitous and abundant members of soil bacterial communities. In this work, class *Acidobacteria-6* was one of the most abundant taxa in those soil profiles (Supplementary Figure S5A), which was in line with the core microbiota in vineyard soils in America (Zarraonaindia et al., 2015). Jones et al. (2009) found that acidobacterial phylotype no. 32 contributed to soil biochemistry, however, its detailed ecological roles remained elusive. Additionally, the member of *Actinobacteria*, especially *Streptomyces* spp., have been recognized as the producers of many soluble antibiotics (Loqman et al., 2009). At the genus level, as the dominant taxa (Supplementary Figure S5B), genus *Lactococcus* was related to the malolactic fermentation process in wine (Zarraonaindia et al., 2015; Morgan et al., 2017). Likewise, the relative abundances of *Pseudomonas* and *Bacillus* were lower than another study in France (Martins et al., 2013). It should be noted that *Bacillus* could activate the signaling pathway and iron acquisition machinery to promote the growth of plants (Mendes et al., 2013). In particular, archaeal genus *Candidatus Nitrososphaera*, which has been validated as an ammonia-oxidizing archaea (Pitcher et al., 2010), was also detected with high relative abundance in those soils, indicating that archaea in soils also contributed to the nitrogen transformation (Supplementary Figure S5B). Among fungi, most of the samples, with the exception of sample C3.5, were dominated by phylum *Ascomycota*, which was consistent with previous work in vineyard soils in Italy (Orgiazzi et al., 2012; Castaneda and Barbosa, 2017) and forest soils (Urbanová et al., 2015), indicating the ubiquity and important roles of this phylum. As a member of *Ascomycota*, *Beauveria bassiana* could endophytically colonize grapevine for protecting against putative target pest insects like the vine mealybug *Planococcus ficus* (Rondot and Reineke, 2018). Meanwhile, *Metarhizium*, which might act as biological pest control agents, had high frequency in vineyard soils and was also detected in this work with low abundance (Supplementary Figure S6), indicating the poor resistance of pest (Uzman et al., 2019). Moreover, fungi play pivotal roles in carbon, nitrogen and phosphorus cycles in soil, as well as wine *terroir* (Klaubauf et al., 2010; Bokulich et al., 2014). *Tetracladium*, which was the most abundant fungal genus found in this work (Supplementary Figure S5C), is long known as plant endophyte and contribute to plant debris degradation (Klaubauf et al., 2010). Unfortunately, genera *Monographella* and *Fusarium*, previously described as the potentially phytopathogenic fungi (Supplementary Figure S6), were also detected with high abundance in 20–40 cm soil profiles, indicating the risk of root rot for grapevine (Castaneda and Barbosa, 2017; Wang et al., 2018).

### Linking Soil Characteristics to Bacterial and Fungal Communities

In general, soil microbial communities can be extensively mediated by management (Orgiazzi et al., 2012; Burns et al., 2016; Holland et al., 2016), geographic distances (Burns et al., 2015), soil depth (Blume et al., 2002; Griffiths et al., 2003; Eilers et al., 2012) and soil properties (Allison et al., 2007). Indeed, BEST analysis showed that soil pH, SOC content, C/N ratio and TP played pivotal roles in determining the soil bacterial assemblies (*R* = 0.494, *P* = 0.001), while only SOC content was important in shaping fungal community (*R* = 0.443, *P* = 0.001). As a proxy for changing soil edaphic factors, especially SOC contents in the 0–20 cm soil profiles, soil depth negatively drove the SOC contents, which was one of the major drivers of microbial community structure among the four sites examined over depth (Table 1 and Figure 3). Genera *Lactococcus* and *Pseudomonas* exhibited negative correlations with SOC contents (Figure 4A), indicating their heterotrophic characteristics in metabolism and ecological function. As for fungal community, genus *Tetracladium*, which was more abundant in soil samples from site C, possessed significantly positive associations with SOC contents and amylase activity (Figure 4B), since *Tetracladium* sp. could secrete a novel cold-adapted glucoamylase (Carrasco et al., 2017). Genus *Geomyces*, a plant-beneficial fungal, could produce cold-adapted amylase and showed positive correlations with amylase and invertase activities, which was consistent with the work by Si et al. (2018). Additionally, soil pH was the most crucial factors in explaining the bacterial community variation (Figure 3A), which was in accordance with previous studies in vineyard soils (Burns et al., 2015) and agricultural landscape soils (Constancias et al., 2015). Meanwhile, *Lactococcus*, which was ubiquitous in soil environmental, mostly distributed in 20–40 cm soils and exhibited significantly negative association with TN (Figure 4A and Supplementary Figure S5B), since members of *Lactococcus* was capable of nitrite and nitrate under anaerobic conditions (Yun et al., 2007). Similarly, *Pseudomonas*, which has been reported as denitrifiers (Kim et al., 2008), was most abundant in soils and behaved in negative correlation with TN (Figure 4A and Supplementary Figure S5B). Together, the results of this work confirmed that microbial community structure was mostly determined by environmental heterogeneity, which occurred even at a small range.

## Conclusion

In the present work, soil bacterial and fungal communities were significantly mediated by edaphic properties, indicating the environmental heterogeneity even at a small range of depth (0–40 cm) and intra-vineyard samples. Among them, pH and SOC content were important factors in shaping bacterial and fungal community structure, respectively. Bacterial diversity within vineyard soil was significantly higher than fungal diversity, possibly indicating that bacteria played more pivotal roles in vineyard soil than fungi. Distribution patterns of individual fungal taxa reflected the potential risk in grapevine disease. Thus, this work could provide guidelines for vineyard soil management and grapevine disease prevention.

## Author Contributions

LL designed the experiments. JY and XW performed the experiments and analyzed the data. HL, XW, and LL wrote the manuscript. All authors read and approved the manuscript.

## Conflict of Interest Statement

The authors declare that the research was conducted in the absence of any commercial or financial relationships that could be construed as a potential conflict of interest.

## References

[B1] Allison V. J. Condron L. M. Peltzer D. A. Richardson S. J. Turner B. L. (2007). Changes in enzyme activities and soil microbial community composition along carbon and nutrient gradients at the franz josef chronosequence. *N. Zeal. Soil Biol. Biochem.* 39 1770–1781. 10.1016/j.soilbio.2007.02.006

[B2] Baddam R. Reddy G. B. Raczkowski C. Cyrus J. S. (2016). Activity of soil enzymes in constructed wetlands treated with swine wastewater. *Ecol. Eng.* 91 24–30. 10.1016/j.ecoleng.2016.01.021

[B3] Blume E. Bischoff M. Reichert J. M. Moorman T. Konopka A. Turco R. F. (2002). Surface and subsurface microbial biomass, community structure and metabolic activity as a function of soil depth and season. *Appl. Soil Ecol.* 20 171–181. 10.1016/S0929-1393(02)00025-2

[B4] Bokulich N. A. Thorngate J. H. Richardson P. M. Mills D. A. (2014). Microbial biogeography of wine grapes is conditioned by cultivar, vintage, and climate. *Proc. Natl. Acad. Sci. U.S.A.* 111 E139–E148. 10.1073/pnas.1317377110 24277822 PMC3890796

[B5] Burns K. N. Bokulich N. A. Cantu D. Greenhut R. F. Kluepfel D. A. O’geen A. T. (2016). Vineyard soil bacterial diversity and composition revealed by 16S rRNA genes: differentiation by vineyard management. *Soil Biol. Biochem.* 103 337–348. 10.1016/j.soilbio.2016.09.007

[B6] Burns K. N. Kluepfel D. A. Strauss S. L. Bokulich N. A. Cantu D. Steenwerth K. L. (2015). Vineyard soil bacterial diversity and composition revealed by 16S rRNA genes: differentiation by geographic features. *Soil Biol. Biochem.* 91 232–247. 10.1016/j.soilbio.2015.09.002

[B7] Canfora L. Vendramin E. Felici B. Tarricone L. Florio A. Benedetti A. (2018). Vineyard microbiome variations during different fertilisation practices revealed by 16s rRNA gene sequencing. *Appl. Soil Ecol.* 125 71–80. 10.1016/j.apsoil.2017.12.019

[B8] Caporaso J. G. Kuczynski J. Stombaugh J. Bittinger K. Bushman F. D. Costello E. K. (2010). QIIME allows analysis of high-throughput community sequencing data. *Nat. Methods* 7 335–336. 10.1038/nmeth.f.303 20383131 PMC3156573

[B9] Caporaso J. G. Lauber C. L. Walters W. A. Berglyons D. Lozupone C. A. Turnbaugh P. J. (2011). Global patterns of 16S rRNA diversity at a depth of millions of sequences per sample. *Proc. Natl. Acad. Sci. U.S.A.* 108 4516–4522. 10.1073/pnas.1000080107 20534432 PMC3063599

[B10] Carrasco M. Alcaino J. Cifuentes V. Baeza M. (2017). Purification and characterization of a novel cold adapted fungal glucoamylase. *Microb. Cell Fact.* 16:75. 10.1186/s12934-017-0693-x 28464820 PMC5414198

[B11] Castaneda L. E. Barbosa O. (2017). Metagenomic analysis exploring taxonomic and functional diversity of soil microbial communities in chilean vineyards and surrounding native forests. *PeerJ* 5:e3098. 10.7717/peerj.3098 28382231 PMC5376117

[B12] Chang E. H. Chen T. H. Tian G. Chiu C. Y. (2016). The effect of altitudinal gradient on soil microbial community activity and structure in moso bamboo plantations. *Appl. Soil Ecol.* 98 213–220. 10.1016/j.apsoil.2015.10.018

[B13] Chen X.-L. Wang D. Chen X. Wang J. Diao J.-J. Zhang J.-Y. (2015). Soil microbial functional diversity and biomass as affected by different thinning intensities in a chinese fir plantation. *Appl. Soil Ecol.* 92 35–44. 10.1016/j.apsoil.2015.01.018

[B14] Chen Z. Luo X. Hu R. Wu M. Wu J. Wei W. (2010). Impact of long-term fertilization on the composition of denitrifier communities based on nitrite reductase analyses in a paddy soil. *Microb. Ecol.* 60 850–861. 10.1007/s00248-010-9700-z 20563573

[B15] Cohen G. Dembiec D. Marcus J. (1970). Measurement of catalase activity in tissue extracts. *Anal. Biochem.* 34 30–38. 5440916 10.1016/0003-2697(70)90083-7

[B16] Constancias F. Saby N. P. A. Terrat S. Dequiedt S. Horrigue W. Nowak V. (2015). Contrasting spatial patterns and ecological attributes of soil bacterial and archaeal taxa across a landscape. *MicrobiologyOpen* 4 518–531. 10.1002/mbo3.256 25922908 PMC4475392

[B17] Corneo P. E. Pellegrini A. Cappellin L. Roncador M. Chierici M. Gessler C. (2013). Microbial community structure in vineyard soils across altitudinal gradients and in different seasons. *FEMS Microbiol. Ecol.* 84 588–602. 10.1111/1574-6941.12087 23398556

[B18] de Melo W. J. Marques M. O. Ferreira M. E. De Melo G. M. P. De Melo V. P. (2007). Chemical properties and enzyme activity in a sewage sludge-treated soil. *Commun. Soil Sci. Plant Anal.* 33 1643–1659. 10.1081/css-120004305 21913124

[B19] Deng S. P. Tabatabai M. A. (1994). Colorimetric determination of reducing sugars in soils. *Soil Biol. Biochem.* 26 473–477.

[B20] Eilers K. G. Debenport S. Anderson S. Fierer N. (2012). Digging deeper to find unique microbial communities: the strong effect of depth on the structure of bacterial and archaeal communities in soil. *Soil Biol. Biochem.* 50 58–65. 10.1016/j.soilbio.2012.03.011

[B21] Fernández-Calviño D. Martín A. Arias-Estévez M. Bååth E. Díaz-Raviña M. (2010). Microbial community structure of vineyard soils with different pH and copper content. *Appl. Soil Ecol.* 46 276–282. 10.1016/j.apsoil.2010.08.001

[B22] Gasparatos D. Haidouti C. (2001). A comparison of wet oxidation methods for determination of total phosphorus in soils. *J. Plant Nutr. Soil Sci.* 164 435–439.

[B23] Griffiths R. I. Whiteley A. S. O’donnell A. G. Bailey M. J. (2003). Influence of depth and sampling time on bacterial community structure in an upland grassland soil. *FEMS Microbiol. Ecol.* 43 35–43. 10.1111/j.1574-6941.2003.tb01043.x 19719694

[B24] Holland T. C. Bowen P. A. Bogdanoff C. P. Lowery T. D. Shaposhnikova O. Smith S. (2016). Evaluating the diversity of soil microbial communities in vineyards relative to adjacent native ecosystems. *Appl. Soil Ecol.* 100 91–103. 10.1016/j.apsoil.2015.12.001

[B25] Huang Y. Kuang Z. Wang W. Cao L. (2016). Exploring potential bacterial and fungal biocontrol agents transmitted from seeds to sprouts of wheat. *Biol. Control* 98 27–33. 10.1016/j.biocontrol.2016.02.013

[B26] Jones R. T. Robeson M. S. Lauber C. L. Hamady M. Knight R. Fierer N. (2009). A comprehensive survey of soil acidobacterial diversity using pyrosequencing and clone library analyses. *ISME J.* 3 442–453. 10.1038/ismej.2008.127 19129864 PMC2997719

[B27] Kandeler E. Gerber H. (1988). Short-term assay of soil urease activity using colorimetric determination of ammonium. *Biol. Fertil. Soils* 6 68–72.

[B28] Kim M. Jeong S. Y. Yoon S. J. Cho S. J. Kim Y. H. Kim M. J. (2008). Aerobic denitrification of *Pseudomonas putida* AD-21 at different C/N ratios. *J. Biosci. Bioeng.* 106 498–502. 10.1263/jbb.106.498 19111647

[B29] Klaubauf S. Inselsbacher E. Zechmeister-Boltenstern S. Wanek W. Gottsberger R. Strauss J. (2010). Molecular diversity of fungal communities in agricultural soils from lower austria. *Fungal Divers.* 44 65–75. 10.1007/s13225-010-0053-1 23794962 PMC3688302

[B30] Li P. Lin W. Liu X. Wang X. Gan X. Luo L. (2017). Effect of bioaugmented inoculation on microbiota dynamics during solid-state fermentation of daqu starter using autochthonous of *Bacillus*, *Pediococcus*, *Wickerhamomyces* and *Saccharomycopsis*. *Food Microbiol.* 61 83–92. 10.1016/j.fm.2016.09.004 27697173

[B31] Li Y. Bezemer T. M. Yang J. Lü X. Li X. Liang W. (2019). Changes in litter quality induced by N deposition alter soil microbial communities. *Soil Biol. Biochem.* 130 33–42. 10.1016/j.soilbio.2018.11.025

[B32] Liu J. Sui Y. Yu Z. Shi Y. Chu H. Jin J. (2014). High throughput sequencing analysis of biogeographical distribution of bacterial communities in the black soils of northeast China. *Soil Biol. Biochem.* 70 113–122. 10.1016/j.soilbio.2013.12.014

[B33] Loqman S. Barka E. A. Clément C. Ouhdouch Y. (2009). Antagonistic actinomycetes from moroccan soil to control the grapevine gray mold. *World J. Microbiol. Biotechnol.* 25 81–91. 10.1007/s11274-008-9864-6

[B34] Martins G. Lauga B. Miot-Sertier C. Mercier A. Lonvaud A. Soulas M. L. (2013). Characterization of epiphytic bacterial communities from grapes, leaves, bark and soil of grapevine plants grown, and their relations. *PLoS One* 8:e73013. 10.1371/journal.pone.0073013 24023666 PMC3758280

[B35] Mendes R. Garbeva P. Raaijmakers J. M. (2013). The rhizosphere microbiome: significance of plant beneficial, plant pathogenic, and human pathogenic microorganisms. *FEMS Microbiol. Rev.* 37 634–663. 10.1111/1574-6976.12028 23790204

[B36] Mezzasalma V. Sandionigi A. Guzzetti L. Galimberti A. Grando M. S. Tardaguila J. (2018). Geographical and cultivar features differentiate grape microbiota in northern italy and spain vineyards. *Front. Microbiol.* 9:946. 10.3389/fmicb.2018.00946 29867854 PMC5962658

[B37] Miguéns T. Leirós M. A. Gil-Sotres F. Trasar-Cepeda C. (2007). Biochemical properties of vineyard soils in galicia. *Spain Sci. Total Environ.* 378 218–222. 10.1016/j.sciotenv.2007.01.050 17316764

[B38] Morgan H. H. Du Toit M. Setati M. E. (2017). The grapevine and wine microbiome: insights from high-throughput amplicon sequencing. *Front. Microbiol.* 8:820. 10.3389/fmicb.2017.00820 28553266 PMC5425579

[B39] Muyzer G. Waal E. C. D. Uitterlinden G. A. (1993). Profiling of complex microbial populations by denaturing gradient gel electrophoresis analysis of polymerase chain reaction-amplified genes coding for 16S rRNA. *Appl. Environ. Microbiol.* 59 695–700. 7683183 10.1128/aem.59.3.695-700.1993 PMC202176

[B40] Orgiazzi A. Lumini E. Nilsson R. H. Girlanda M. Vizzini A. Bonfante P. (2012). Unravelling soil fungal communities from different mediterranean land-use backgrounds. *PLoS One* 7:e34847. 10.1371/journal.pone.0034847 22536336 PMC3335027

[B41] Pitcher A. Rychlik N. Hopmans E. C. Spieck E. Rijpstra W. I. Ossebaar J. (2010). Crenarchaeol dominates the membrane lipids of *Candidatus Nitrososphaera gargensis*, a thermophilic group I.1b *Archaeon*. *ISME J.* 4 542–552. 10.1038/ismej.2009.138 20033067

[B42] Plaza C. Courtier-Murias D. Fernández J. M. Polo A. Simpson A. J. (2013). Physical, chemical, and biochemical mechanisms of soil organic matter stabilization under conservation tillage systems: a central role for microbes and microbial by-products in C sequestration. *Soil Biol. Biochem.* 57 124–134. 10.1016/j.soilbio.2012.07.026

[B43] Power M. E. Tilman D. Estes J. A. Menge B. A. Bond W. J. Mills L. S. (1996). Challenges in the quest for keystones: identifying keystone species is difficult—but essential to understanding how loss of species will affect ecosystems. *Bioscience* 46 609–620.

[B44] Probst B. Schüler C. Joergensen R. G. (2008). Vineyard soils under organic and conventional management—microbial biomass and activity indices and their relation to soil chemical properties. *Biol. Fertil. Soils* 44 443–450. 10.1007/s00374-007-0225-7

[B45] Qi R. Li J. Lin Z. Li Z. Li Y. Yang X. (2016). Temperature effects on soil organic carbon, soil labile organic carbon fractions, and soil enzyme activities under long-term fertilization regimes. *Appl. Soil Ecol.* 102 36–45. 10.1016/j.apsoil.2016.02.004

[B46] Rondot Y. Reineke A. (2018). Endophytic *Beauveria bassiana* in grapevine *Vitis vinifera* (L.) reduces infestation with piercing-sucking insects. *Biol. Control* 116 82–89. 10.1016/j.biocontrol.2016.10.006

[B47] Si P. Shao W. Yu H. Yang X. Gao D. Qiao X. (2018). Rhizosphere microenvironments of eight common deciduous fruit trees were shaped by microbes in northern china. *Front. Microbiol.* 9:3157. 10.3389/fmicb.2018.03147 30619213 PMC6305578

[B48] Spain A. M. Krumholz L. R. Elshahed M. S. (2009). Abundance, composition, diversity and novelty of soil *Proteobacteria*. *ISME J.* 3 992–1000. 10.1038/ismej.2009.43 19404326

[B49] Steenwerth K. L. Drenovsky R. E. Lambert J. J. Kluepfel D. A. Scow K. M. Smart D. R. (2008). Soil morphology, depth and grapevine root frequency influence microbial communities in a pinot noir vineyard. *Soil Biol. Biochem.* 40 1330–1340. 10.1016/j.soilbio.2007.04.031

[B50] Tabatabai M. A. Bremner J. M. (1969). Use of p -nitrophenyl phosphate for assay of soil phosphatase activity. *Soil Biol. Biochem.* 1 301–307.

[B51] Urbanová M. Šnajdr J. Baldrian P. (2015). Composition of fungal and bacterial communities in forest litter and soil is largely determined by dominant trees. *Soil Biol. Biochem.* 84 53–64. 10.1016/j.soilbio.2015.02.011

[B52] Uzman D. Pliester J. Leyer I. Entling M. H. Reineke A. (2019). Drivers of entomopathogenic fungi presence in organic and conventional vineyard soils. *Appl. Soil Ecol.* 133 89–97. 10.1016/j.apsoil.2018.09.004

[B53] Wang R. Wang Y. Yang Q. Kang C. Li M. (2018). Unraveling the characteristics of the microbial community and potential pathogens in the rhizosphere soil of *Rehmannia glutinosa* with root rot disease. *Appl. Soil Ecol.* 130 271–279. 10.1016/j.apsoil.2018.07.001

[B54] Xu W. Huang Z. Zhang X. Li Q. Lu Z. Shi J. (2011). Monitoring the microbial community during solid-state acetic acid fermentation of zhenjiang aromatic vinegar. *Food Microbiol.* 28 1175–1181. 10.1016/j.fm.2011.03.011 21645817

[B55] Yin-Ru Chiang W. I. (2011). Oxic and anoxic metabolism of steroids by bacteria. *J. Biorem. Biodegrad.* S1:001 10.4172/2155-6199.s1-001

[B56] Yun S. H. Hwang T. S. Park D. H. (2007). Metabolic characterization of lactic acid bacterium *Lactococcus garvieae* sk11, capable of reducing ferric iron, nitrate, and fumarate. *J. Microbiol. Biotechnol.* 17 218–225. 18051752

[B57] Zarraonaindia I. Owens S. M. Weisenhorn P. West K. Hampton-Marcell J. Lax S. (2015). The soil microbiome influences grapevine-associated microbiota. *MBio* 6:e2527-14. 10.1128/mBio.02527-14 25805735 PMC4453523

[B58] Zhang B. Penton C. R. Xue C. Quensen J. F. Roley S. S. Guo J. (2017). Soil depth and crop determinants of bacterial communities under ten biofuel cropping systems. *Soil Biol. Biochem.* 112 140–152. 10.1016/j.soilbio.2017.04.019

