# Corrigendum: Characterizing the Intra-Vineyard Variation of Soil Bacterial and Fungal Communities

**DOI:** 10.3389/fmicb.2019.01551

**Published:** 2019-07-03

**Authors:** Hebin Liang, Xiaowen Wang, Junwei Yan, Lixin Luo

**Affiliations:** ^1^School of Biology and Biological Engineering, South China University of Technology, Guangzhou, China; ^2^Food Testing Institute, Shenzhen Academy of Metrology and Quality Inspection, Shenzhen, China; ^3^National Nutrition Food Testing Center, Shenzhen, China

**Keywords:** vineyard soil, soil characteristics, intra-vineyard scale, bacterial community, fungal community

In the published article, there was an error. The accession numbers for the raw data, deposited in the NCBI Short Read Archive database, are incorrect.

A correction has been made to the **Materials and Methods** section, subsection **Accession Numbers**:

“Raw data in this study have been deposited in the NCBI Short Read Archive database under accession number SRP075187”.

The authors apologize for this error and state that this does not change the scientific conclusions of the article in any way.

