# Supplementary material for: Characterizing the Intra-Vineyard Variation of Soil Bacterial and Fungal Communities
Source: Front Microbiol. 2019 May 31;10:1239. doi: 10.3389/fmicb.2019.01239 (PMC6554343; doi:10.3389/fmicb.2019.01239)
Supplement: Supplementary file 1 [file Data_Sheet_1.docx]

**Supplementary Materials**

**Characterizing the intra-vineyard variation of soil bacterial and fungal communities**

Hebin Liang^a^, Xiaowen Wang^b,c^, Junwei Yan^a^, Lixin Luo^a,*^

^a^School of Biology and Biological Engineering, South China University of Technology, Guangzhou, 510006, PR China

^b^Food Testing Institute, Shenzhen Academy of Metrology & Quality Inspection, Shenzhen, Guangdong, PR China

^c^National Nutrition Food Testing Center, Guangdong

*Corresponding author at: School of Biology and Biological Engineering, South China University of Technology Guangzhou higher education Megacenter, Panyu, Guangzhou, 510006, Guangdong, China

E-mail address: [btlxluo@scut.edu.cn](mailto:btlxluo@scut.edu.cn) (Lixin Luo).

Note: Hebin Liang and Xiaowen Wang contributed equally to this work.

**Summary**

Table S1 The enzyme activities of different soil samples. The enzyme activities for amylase, invertase, urease, catalase and phosphatase were calculated in units of mg maltose g^-1^ h^-1^, mg glucose g^-1^ h^-1^, mg NH_4_^+^-N g^-1^ 24 h^-1^, 0.02M KMnO_4_ ml g^-1^ and mg phenol 100 g^-1^, respectively.

Table S2 Observed 16S rRNA and ITS1 rRNA sequencing results and α-diversity indices in vineyard soil samples.

Table S3 Spearman correlation coefficients for relationships between soil physicochemical properties and bacterial diversity.

Table S4 Spearman correlation coefficients for relationships between soil physicochemical properties and fungal diversity.

Figure S1 Abundances of bacterial 16S rDNA (red) and fungal 18S rDNA (blue) in all depths and sites.

Figure S2 Rarefaction curves of bacteria (A) and fungi (B) at 3% dissimilarity in vineyard soils.

Figure S3 Comparisons of bacterial (A, B) and fungal(C, D) Chao1 indexes among soil depths (A, C) and among sites (B, D).

Figure S4 Cluster analysis of bacterial (A) and fungal (B) communities in the 16 samples based on Weighted UniFrac matrix.

Figure S5 Relative abundance of top 10 bacterial class (A), bacterial genus (B) and fungal genus(C).

Figure S6 Heatmap shows the top 35 fungal genus composition of all samples. The heatmap was generated on the standardized value (z-score) of individual relative abundances.

Bioinformatics scripts in this work

Table S1 The enzyme activities of different soil samples. The enzyme activities for amylase, invertase, urease, catalase and phosphatase were calculated in units of mg maltose g^-1^ h^-1^, mg glucose g^-1^ h^-1^, mg NH_4_^+^-N g^-1^ 24 h^-1^, 0.02M KMnO_4_ ml g^-1^ and mg phenol 100 g^-1^, respectively.

| Sample | Amylase | Invertase | Urease | Catalase | Phosphatase |
| --- | --- | --- | --- | --- | --- |
| A1.5 | 2.31±0.119 | 4.01±0.319 | 0.16±0.0013 | 14.11±0.63 | 29.81±0.51 |
| A1.10 | 5.28±0.152 | 2.85±0.152 | 0.14±0.0033 | 16.16±0.91 | 22.17±2.27 |
| A1.20 | 1.93±0.155 | 0.75±0.155 | 0.14±0.0044 | 15.81±1.08 | 3.70±0.31 |
| A1.40 | 1.89±0.111 | 0.61±0.111 | 0.12±0.0041 | 19.49±1.11 | 1.82±0.16 |
| B2.5 | 3.9±0.179 | 7.18±0.379 | 0.15±0.0028 | 18.11±0.65 | 20.78±1.31 |
| B2.10 | 4.51±0.152 | 5.18±0.252 | 0.14±0.0035 | 18.9±1.98 | 9.51±1.06 |
| B2.20 | 2.77±0.19 | 4.29±0.19 | 0.14±0.0026 | 17.78±0.96 | 7.03±0.61 |
| B2.40 | 2.62±0.141 | 3.52±0.141 | 0.14±0.0034 | 18.11±0.94 | 4.55±0.21 |
| C3.5 | 5.28±0.127 | 5.17±0.127 | 0.17±0.0059 | 15.03±1.01 | 29.67±1.03 |
| C3.10 | 5.62±0.15 | 3.79±0.15 | 0.15±0.004 | 16.47±0.80 | 19.71±1.01 |
| C3.20 | 4.86±0.138 | 3.01±0.138 | 0.13±0.005 | 16.25±0.79 | 8.99±0.60 |
| C3.40 | 3.27±0.16 | 2.39±0.16 | 0.12±0.0021 | 16.66±0.81 | 4.65±0.32 |
| D4.5 | 2.83±0.127 | 5.36±0.127 | 0.14±0.0017 | 13.46±0.97 | 28.65±0.93 |
| D4.10 | 4.72±0.15 | 4.04±0.15 | 0.14±0.0028 | 16.47±1.18 | 18.56±0.92 |
| D4.20 | 2.45±0.118 | 0.9±0.118 | 0.13±0.0018 | 4.34±0.74 | 8.39±0.17 |
| D4.40 | 2.42±0.14 | 0.45±0.14 | 0.12±0.0039 | 17.53±0.99 | 2.66±0.09 |

Table S2 Observed 16S rRNA and ITS1 rRNA sequencing results and α-diversity indices in vineyard soil samples.

| Samples | No.of | | α-diversity (97%) | | | | | |
| --- | --- | --- | --- | --- | --- | --- | --- | --- |
|  | Raw tags | Effective tags | Observed species | Shannon | Simpson | Chao1 | ACE | Goods coverage |
| **16S** |  |  |  |  |  |  |  |  |
| A1.5 | 34455 | 33585 | 2265 | 9.087 | 0.992 | 2616.12 | 2722.79 | 0.978 |
| A1.10 | 58612 | 55348 | 2492 | 9.050 | 0.993 | 3506.66 | 3611.52 | 0.964 |
| A1.20 | 66759 | 65443 | 2593 | 8.987 | 0.990 | 3280.89 | 3507.30 | 0.967 |
| A1.40 | 52928 | 51447 | 2379 | 8.409 | 0.984 | 3405.15 | 3587.57 | 0.964 |
| B2.5 | 27750 | 27044 | 2211 | 9.191 | 0.994 | 2352.54 | 2466.25 | 0.985 |
| B2.10 | 55516 | 54312 | 2518 | 9.272 | 0.995 | 3132.77 | 3283.80 | 0.970 |
| B2.20 | 54092 | 53024 | 2867 | 9.464 | 0.995 | 3944.80 | 4066.37 | 0.960 |
| B2.40 | 60955 | 59720 | 2843 | 9.449 | 0.994 | 3564.81 | 3725.52 | 0.965 |
| C3.5 | 50261 | 49062 | 3171 | 9.821 | 0.996 | 4275.39 | 4446.07 | 0.956 |
| C3.10 | 61643 | 60166 | 3114 | 9.809 | 0.996 | 3887.73 | 4040.52 | 0.962 |
| C3.20 | 51538 | 50445 | 2926 | 9.349 | 0.993 | 4354.00 | 4417.52 | 0.955 |
| C3.40 | 55815 | 54655 | 2473 | 9.340 | 0.995 | 3128.74 | 3209.08 | 0.971 |
| D4.5 | 50247 | 49219 | 2733 | 9.406 | 0.993 | 3656.67 | 3764.98 | 0.963 |
| D4.10 | 65674 | 64367 | 2547 | 9.097 | 0.991 | 3227.27 | 3406.02 | 0.968 |
| D4.20 | 66124 | 64752 | 2352 | 8.754 | 0.990 | 3042.25 | 3228.51 | 0.969 |
| D4.40 | 51759 | 50605 | 2398 | 8.671 | 0.989 | 3369.03 | 3513.78 | 0.965 |
| **ITS** |  |  |  |  |  |  |  |  |
| A1.5 | 59065 | 57988 | 330 | 3.386 | 0.813 | 453.051 | 476.95 | 0.996 |
| A1.10 | 56916 | 55617 | 203 | 1.504 | 0.346 | 323.677 | 341.077 | 0.997 |
| A1.20 | 69680 | 68160 | 376 | 2.912 | 0.589 | 505.045 | 520.574 | 0.996 |
| A1.40 | 53179 | 49327 | 440 | 4.148 | 0.874 | 548.896 | 557.829 | 0.996 |
| B2.5 | 71586 | 69958 | 224 | 1.878 | 0.425 | 294.435 | 327.84 | 0.997 |
| B2.10 | 62692 | 61950 | 322 | 1.835 | 0.400 | 492.66 | 519.946 | 0.996 |
| B2.20 | 62888 | 61346 | 327 | 2.655 | 0.718 | 474.873 | 532.094 | 0.996 |
| B2.40 | 69231 | 66730 | 788 | 5.546 | 0.890 | 954.703 | 982.409 | 0.993 |
| C3.5 | 57956 | 35855 | 271 | 2.851 | 0.698 | 317.887 | 325.232 | 0.998 |
| C3.10 | 64952 | 63421 | 362 | 1.866 | 0.364 | 524.559 | 533.16 | 0.996 |
| C3.20 | 72403 | 69074 | 324 | 1.735 | 0.337 | 417.059 | 459.85 | 0.996 |
| C3.40 | 61083 | 57651 | 345 | 1.802 | 0.364 | 447.514 | 488.868 | 0.996 |
| D4.5 | 65693 | 64147 | 485 | 4.899 | 0.920 | 625.759 | 671.493 | 0.995 |
| D4.10 | 74874 | 72334 | 459 | 4.885 | 0.913 | 576.692 | 598.894 | 0.996 |
| D4.20 | 56939 | 53471 | 708 | 6.083 | 0.967 | 910.843 | 861.377 | 0.994 |
| D4.40 | 34094 | 32496 | 603 | 5.172 | 0.930 | 1502.583 | 977.568 | 0.992 |

Table S3 Spearman correlation coefficients for relationships between soil physicochemical properties and bacterial diversity. Number in bold indicates the significant correlation.

| Spearman | Observed species | Shannon | Chao1 | Moisture | pH | SOC | TN | C/N | TP | AP |
| --- | --- | --- | --- | --- | --- | --- | --- | --- | --- | --- |
| Observed species | 1 |  |  |  |  |  |  |  |  |  |
| Shannon | **0.87** | 1 |  |  |  |  |  |  |  |  |
| Chao1 | **0.98** | **0.84** | 1 |  |  |  |  |  |  |  |
| Moisture | -0.23 | -0.03 | -0.25 | 1 |  |  |  |  |  |  |
| pH | **-0.72** | **-0.74** | **-0.72** | -0.28 | 1 |  |  |  |  |  |
| SOC | 0.19 | 0.33 | 0.20 | **0.83** | **-0.70** | 1 |  |  |  |  |
| TN | 0.13 | 0.31 | 0.13 | **0.87** | **-0.62** | **0.98** | 1 |  |  |  |
| C/N | 0.45 | **0.61** | 0.43 | 0.31 | **-0.65** | **0.62** | 0.57 | 1 |  |  |
| TP | -0.57 | -0.33 | **-0.64** | 0.46 | 0.55 | 0.03 | 0.17 | -0.04 | 1 |  |
| AP | -0.15 | 0.09 | -0.18 | **0.81** | -0.20 | **0.69** | **0.72** | **0.62** | 0.56 | 1 |

Table S4 Spearman correlation coefficients for relationships between soil physicochemical properties and fungal diversity. Number in bold indicates the significant correlation.

| Spearman | Observed species | Shannon | Chao1 | Moisture | pH | SOC | TN | C/N | TP | AP |
| --- | --- | --- | --- | --- | --- | --- | --- | --- | --- | --- |
| Observed species | 1 |  |  |  |  |  |  |  |  |  |
| Shannon | **0.66** | 1 |  |  |  |  |  |  |  |  |
| Chao1 | **0.86** | **0.85** | 1 |  |  |  |  |  |  |  |
| Moisture | -0.52 | **-0.79** | **-0.72** | 1 |  |  |  |  |  |  |
| pH | -0.29 | 0.22 | -0.06 | -0.22 | 1 |  |  |  |  |  |
| SOC | -0.39 | **-0.79** | **-0.65** | **0.91** | -0.54 | 1 |  |  |  |  |
| TN | -0.41 | **-0.79** | **-0.66** | **0.93** | -0.51 | **0.99** | 1 |  |  |  |
| C/N | -0.44 | -0.41 | -0.48 | **0.60** | -0.50 | **0.75** | **0.72** | 1 |  |  |
| TP | **-0.77** | -0.44 | **-0.68** | **0.58** | 0.54 | 0.30 | 0.34 | 0.28 | 1 |  |
| AP | **-0.61** | -0.53 | **-0.65** | **0.82** | -0.09 | **0.76** | **0.77** | **0.80** | **0.68** | 1 |


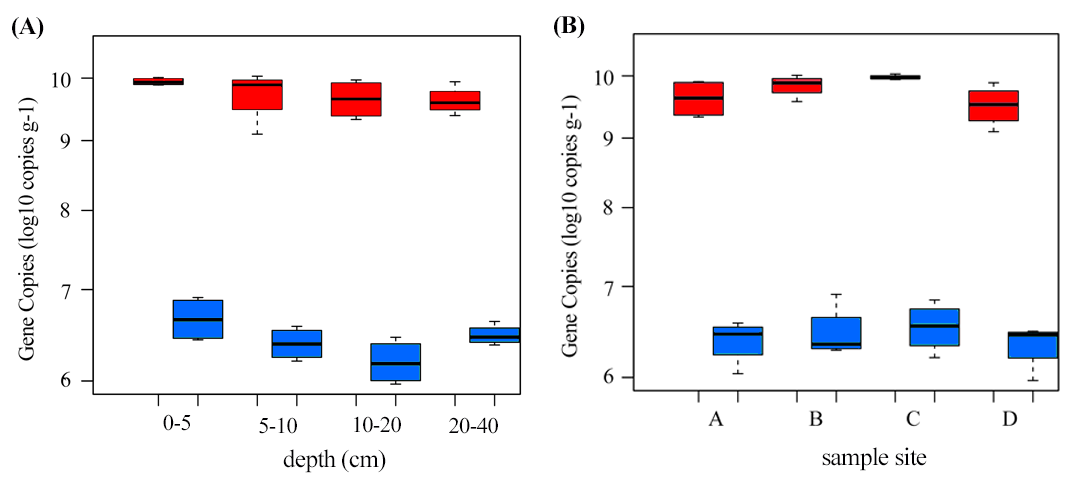


Figure S1 Abundances of bacterial 16S rDNA (red) and fungal 18S rDNA (blue) in all depths and sites.


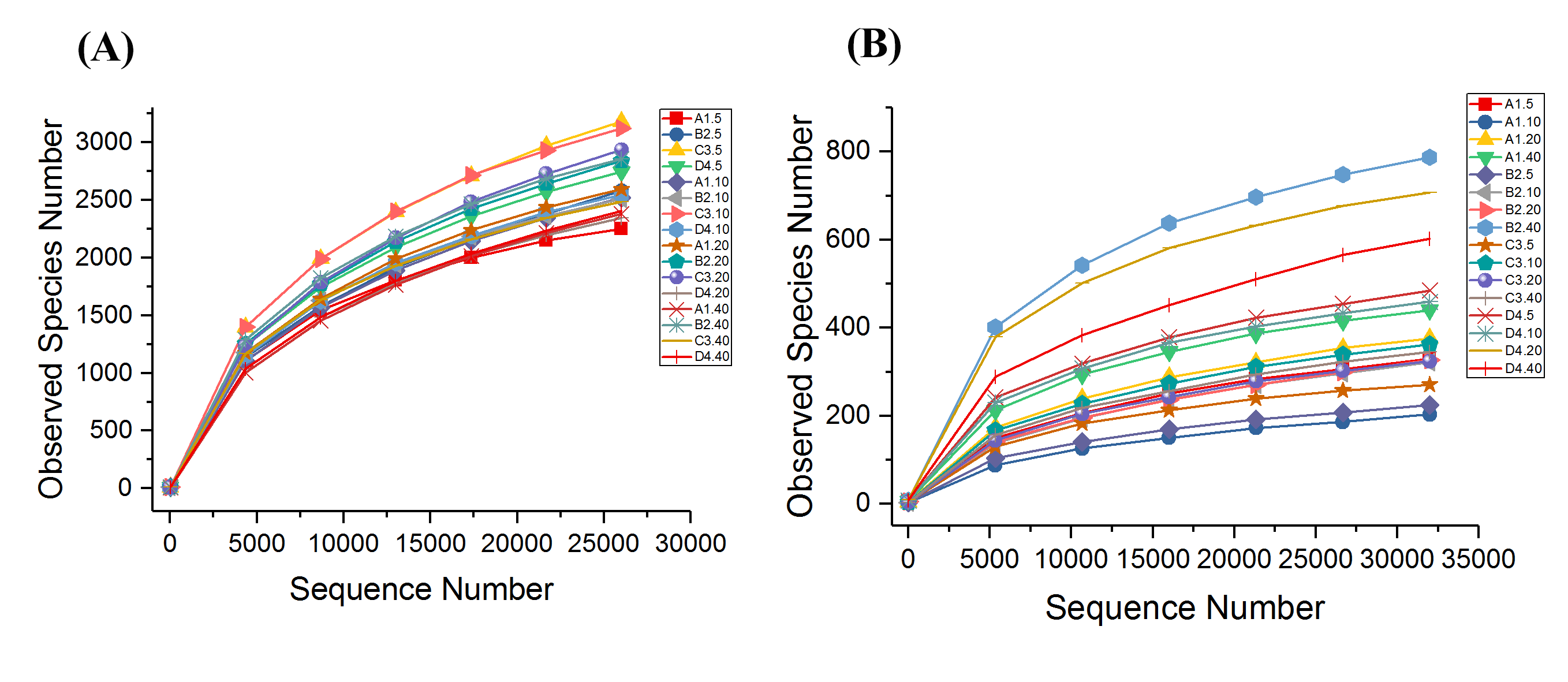


Figure S2 Rarefaction curves of bacteria (A) and fungi (B) at 3% dissimilarity in vineyard soils.


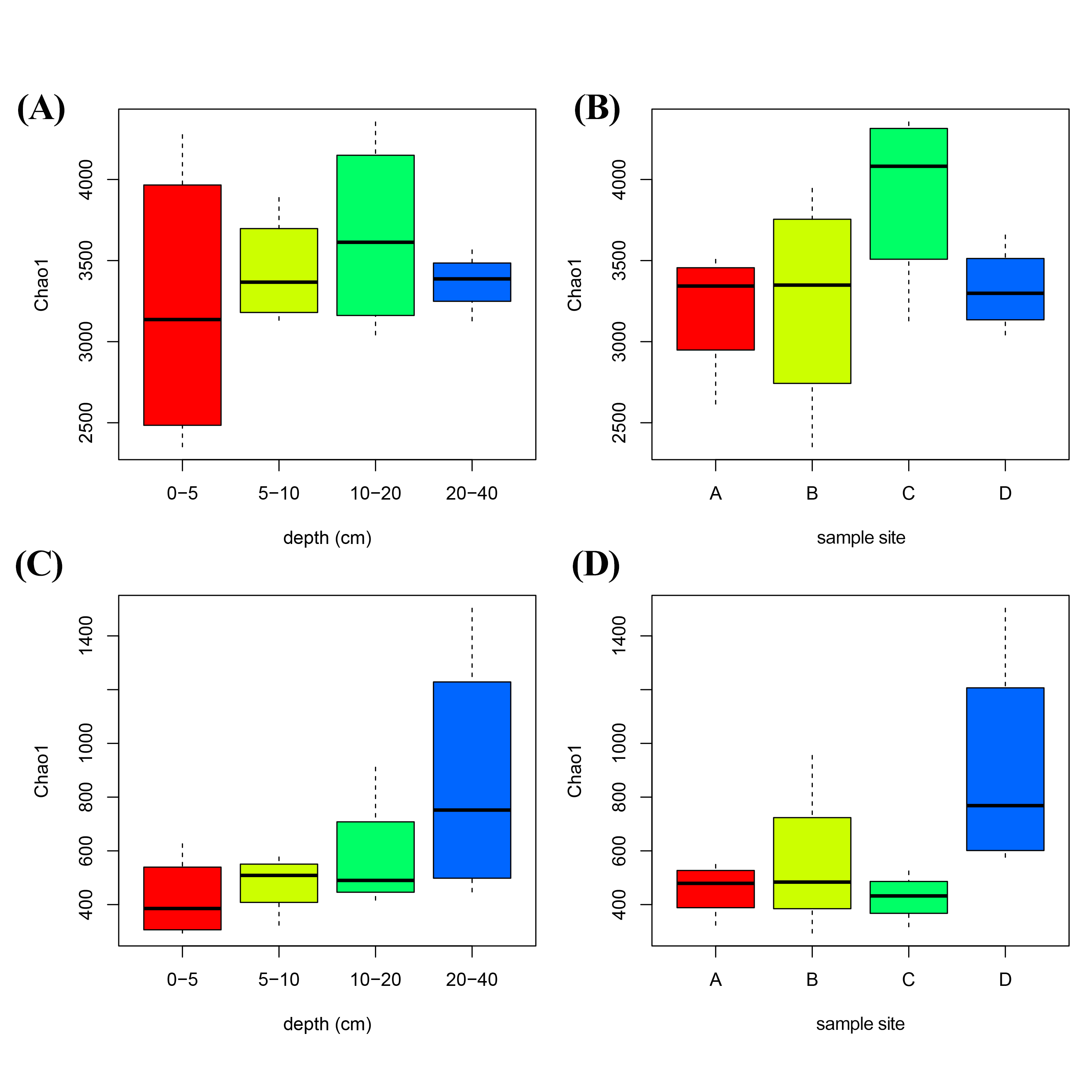


Figure S3 Comparisons of bacterial (A, B) and fungal(C, D) Chao1 indexes among soil depths (A, C) and among sites (B, D).


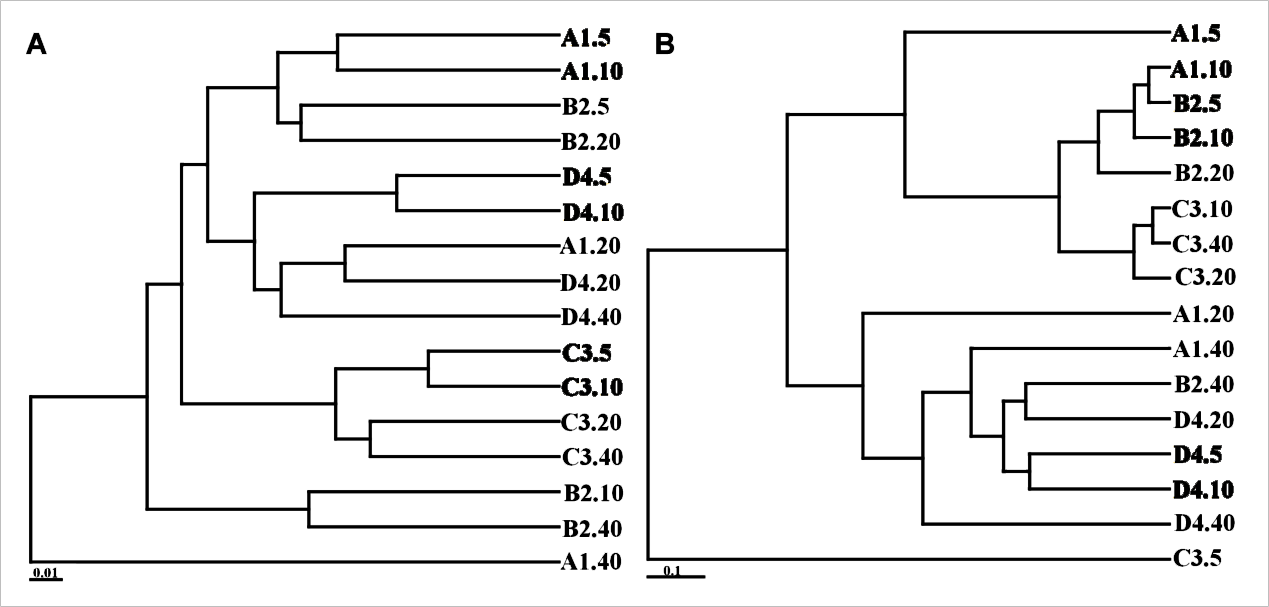


Figure S4 Cluster analysis of bacterial (A) and fungal (B) communities in the 16 samples based on Weighted UniFrac matrix.


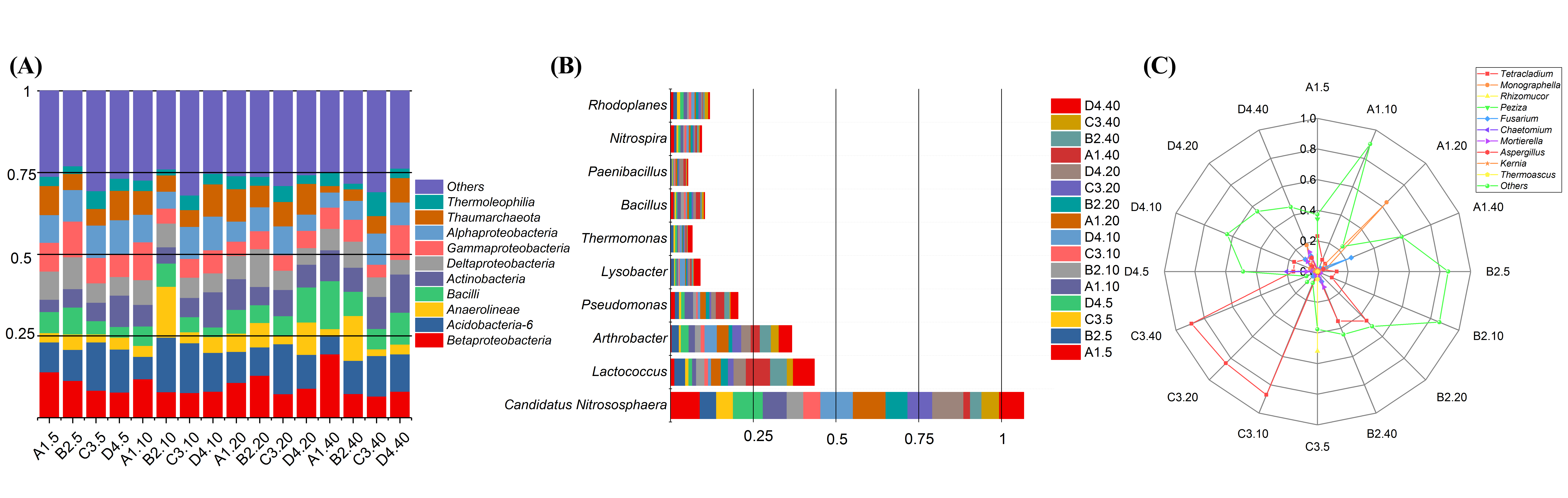


Figure S5 Relative abundance of top 10 bacterial class (A), bacterial genus (B) and fungal genus(C).


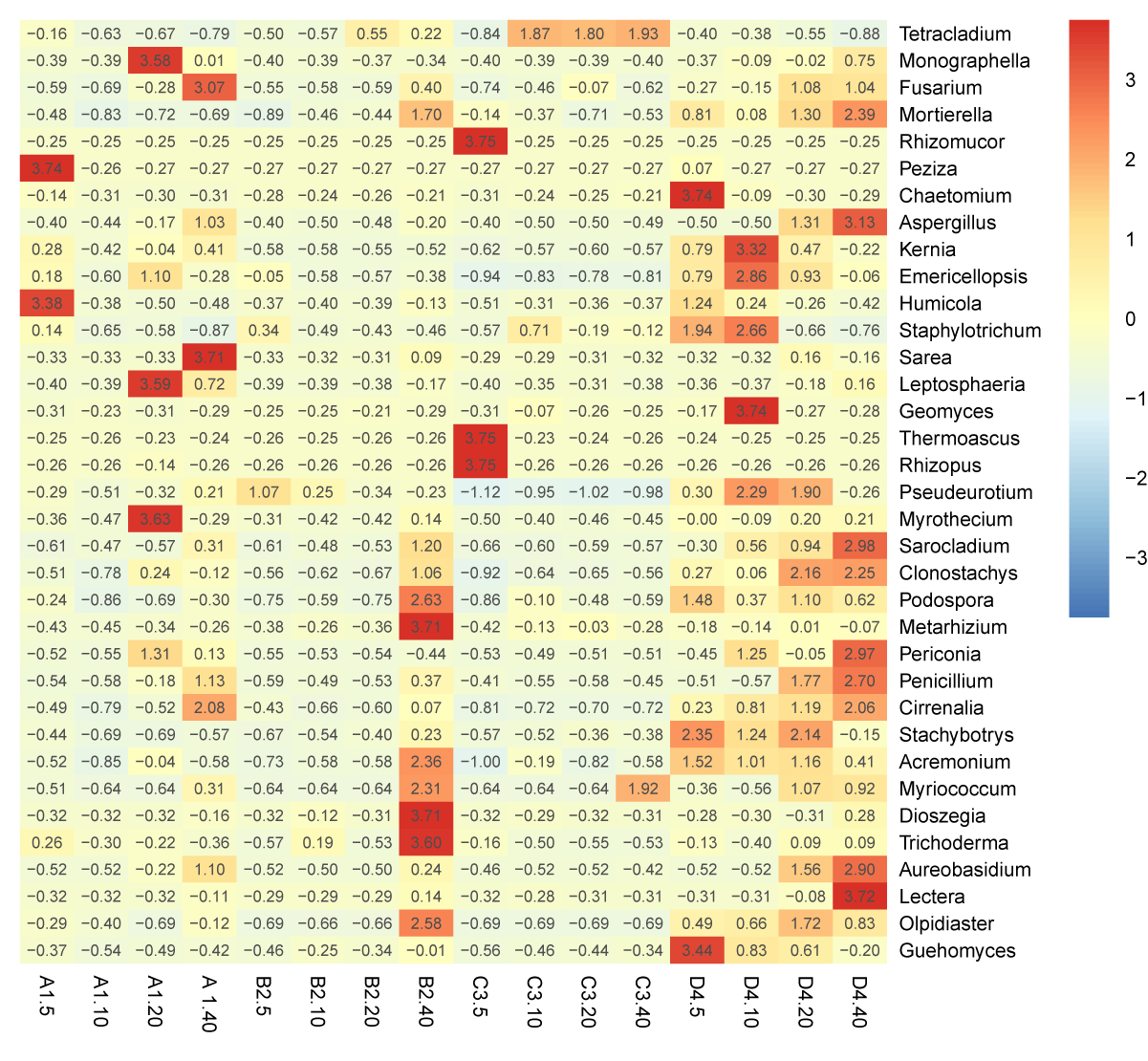


Figure S6 Heatmap shows the top 35 fungal genus composition of all samples. The heatmap was generated on the standardized value (z-score) of individual relative abundances.

**Bioinformatics scripts in this work**

**# Mantel test**

library(vegan)

veg.dist <- vegdist(spec,method="bray")

env.dist <- vegdist(env, method="bray")

mantel(veg.dist, env.dist, method="spear")

**# Permutational multivariate analysis of variance (PERMANOVA)**

library(vegan)

adonis(formula=spe~ group,data = group,permutations = 999,method="bray")

**# Principal coordinates analysis (PCoA)**

library(ape)

data <- pcoa(matrix)

data$values

biplot(data)

**# Cluster analysis**

data <- hclust(spe.dist,method="complete")

plot(data,hang=-1)

**# BEST**

library(vegan)

sol <- bioenv(spec ~ Moisture + pH + SOC + TN + CN+ TP + AP, chem)

sol

summary(sol)

**# Spearman correlation coefficient (SPCC)**

library(Hmisc)

rcorr(matrix, type="spearman")

**# Heatmap**

library(pheatmap)

pheatmap(data,scale="row",show_rownames=T,cluster_rows=F,cluster_cols=F,annotation_legend=T,display_number=T)
